# Supplementary material for: Photosystem II Subunit S overexpression increases the efficiency of water use in a field-grown crop
Source: Nat Commun. 2018 Mar 6;9:868. doi: 10.1038/s41467-018-03231-x (PMC5840416; doi:10.1038/s41467-018-03231-x)
Supplement: Supplementary file 2 — Supplementary Information [file 41467_2018_3231_MOESM2_ESM.docx]

***Photosystem II subunit S* overexpression Iincreases the efficiency of water use in a field-grown crop**

­­­Głowacka *et al*.

**
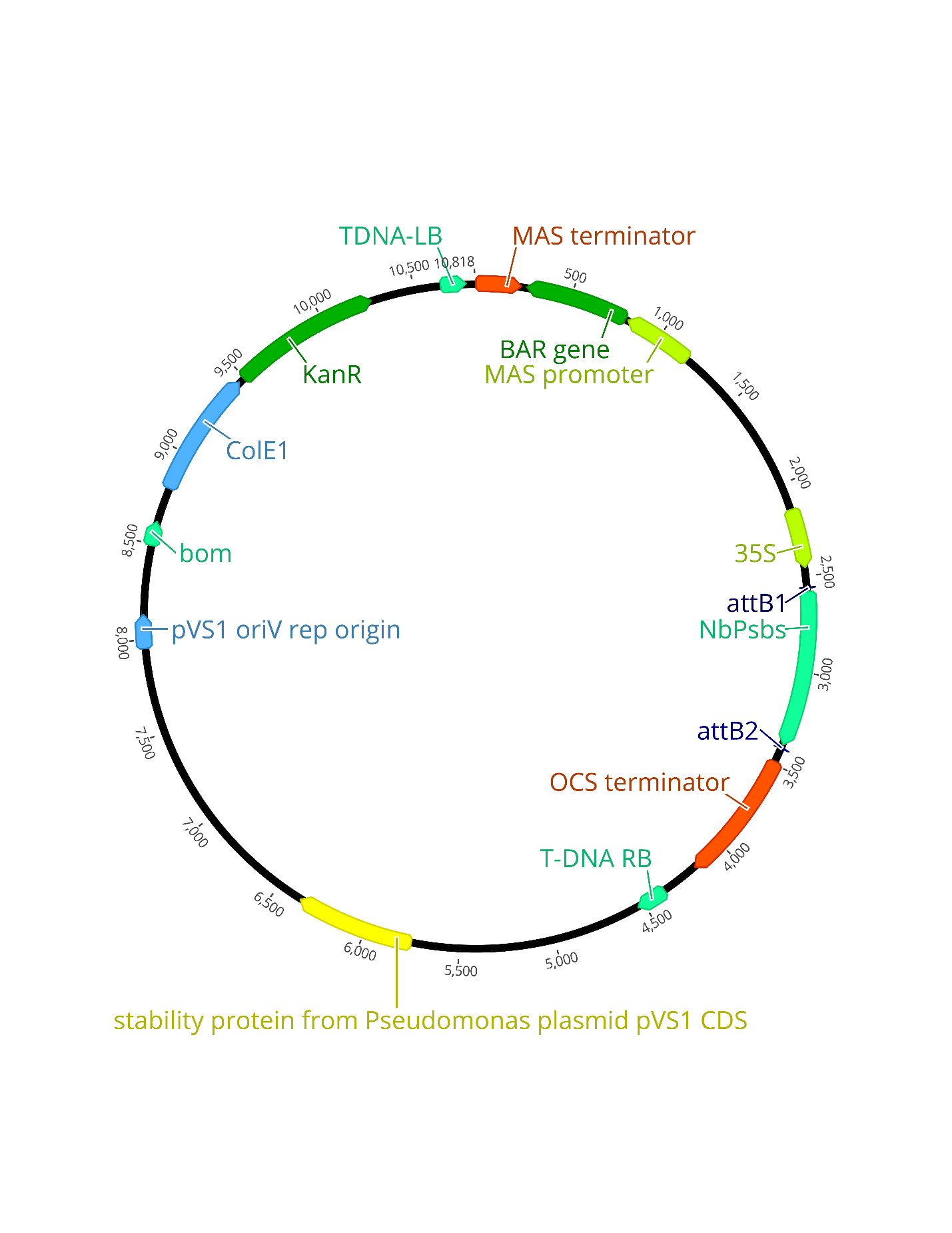
**

**Supplementary Fig. 1** **Plasmid map of construct pEG100-NbPsbS** **used to transform *N. tabacum*.** *NbPsbS* - *Nicotiana bethamiana* Photosystem II subunit S ([www.uniprot.org](http://www.uniprot.org), Q2LAH0_NICBE); BAR - bialaphos resistance; 35S - cauliflower mosaic virus (CaMV) 35S promotor; MAS - mannopine synthase; OCS - octopine synthase; pEarleygate100 was used as the vector backbone.


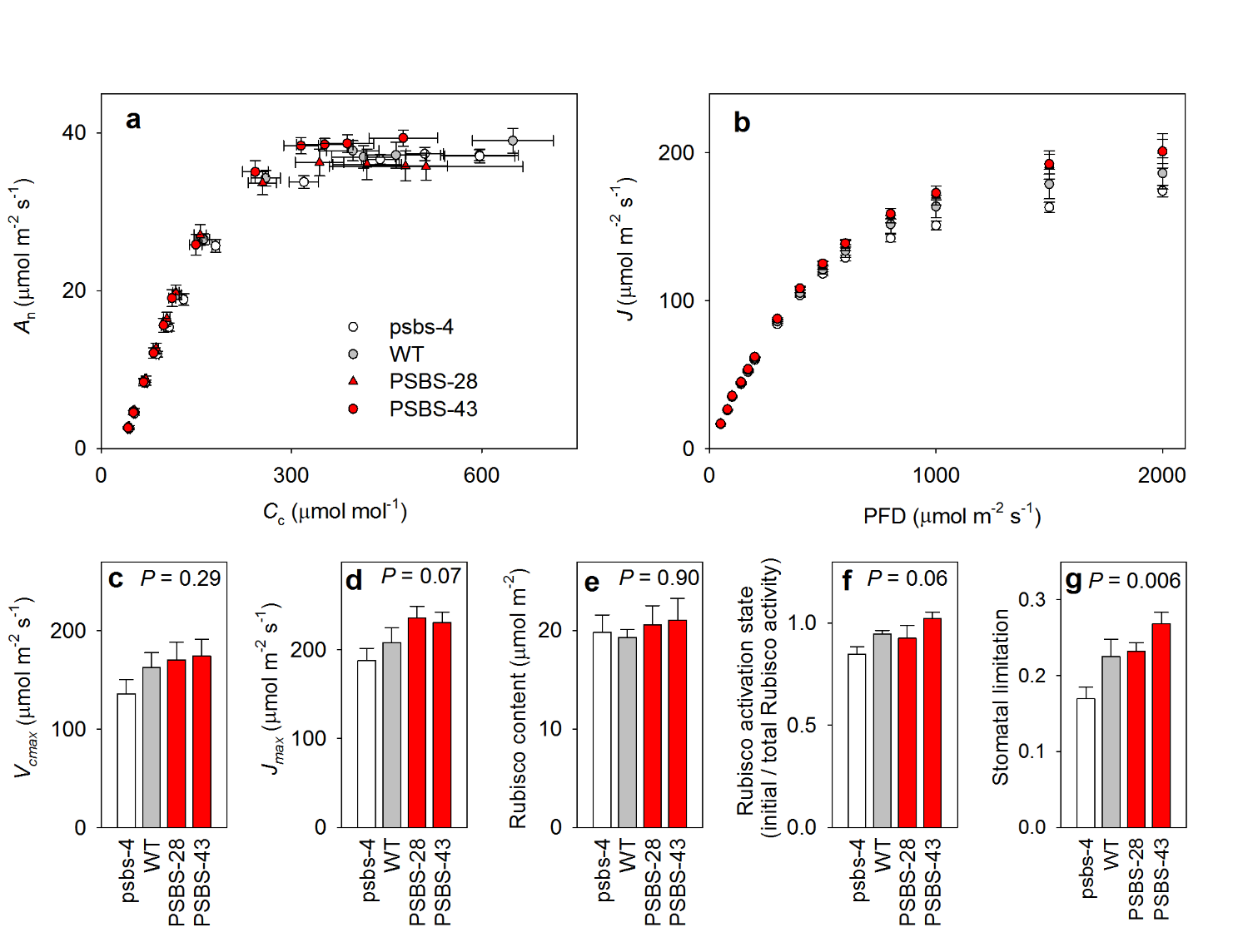


**Supplementary Fig. 2 Biochemical capacity of photosynthesis in *N. tabacum* plants with modified levels of PsbS.**

**(a)** Net CO_2_ fixation rate (*A_n_*) as a function of chloroplastic CO_2_ concentration (*C*_c_); **(b)** Electron transport rate (*J*) as a function of incident light intensity; **(c)** Maximum ribulose bisphosphate carboxylation capacity (*V_cmax_*) and **(d)** Maximum rate of linear electron transport (*J_max_*); **(e)** Rubisco content; **(f)** Rubisco activation state **(g)** stomatal limitation in youngest fully expanded leaves of PsbS-silencing line (psbs-4), PsbS-overexpressing lines (PSBS-28 and PSBS-43), and wild-type (WT) tobacco plants grown under controlled conditions. Error bars show s.e.m. (biological replicates were *n* = from 6 to 10 in panel **a-d** and **g**, *n* = 3 in panel **e,** and *n* = 4 in panel **f**); *P*-values indicate the significance of genotype in ANOVA.

**
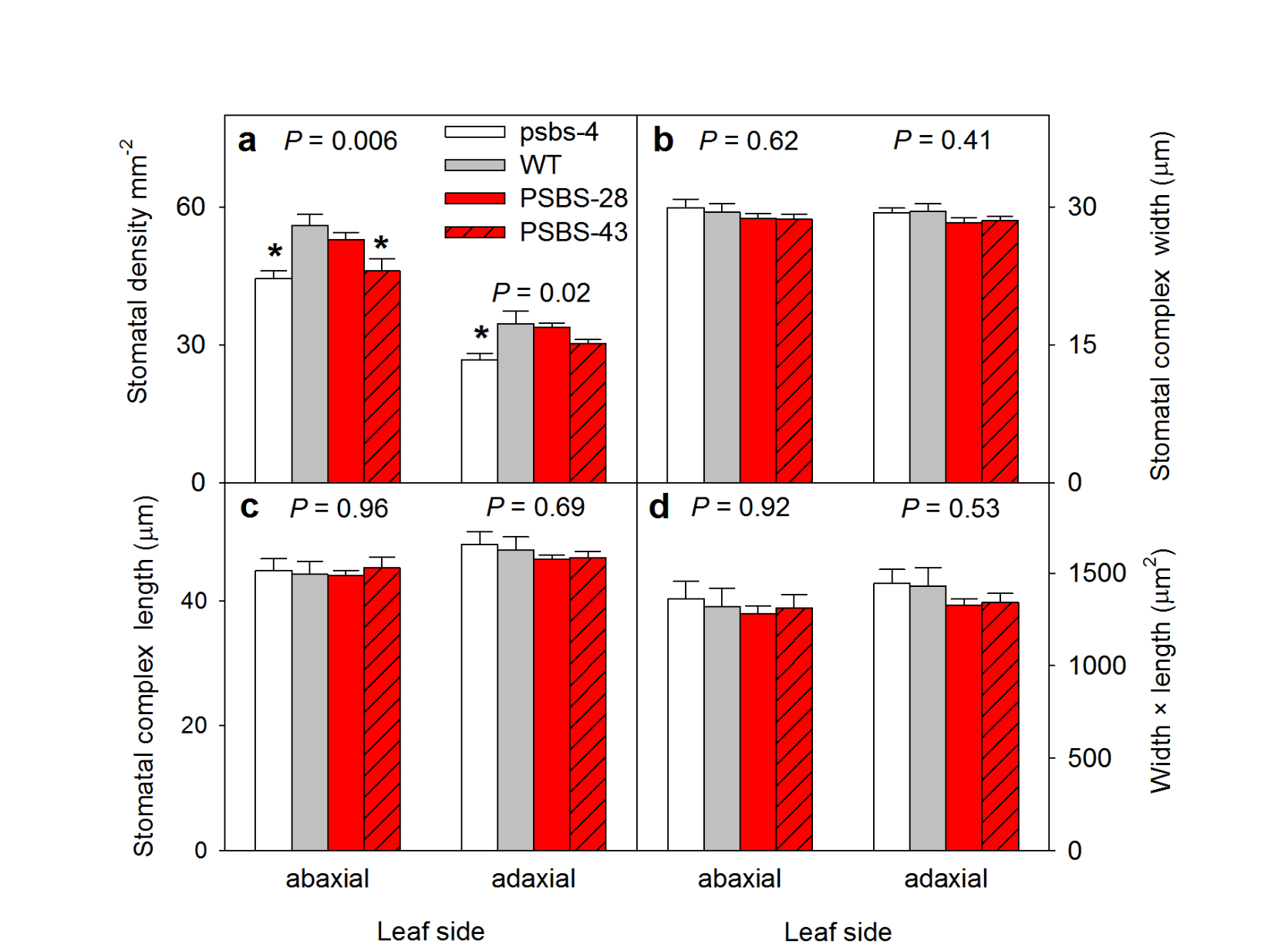
**

**Supplementary Fig. 3** **Stomatal density and dimension** **in *N. tabacum* plants with modified levels of PsbS.**

**(a)** Stomatal density; **(b)** Stomatal complex width; **(c)** Stomatal complex length; **(d)** Stomatal complex width × length in youngest fully expanded leaves of PsbS-silencing line (psbs-4), PsbS-overexpressing lines (PSBS-28 and PSBS-43), and wild-type (WT) tobacco plants grown under controlled conditions. Error bars show s.e.m. (*n* = 4 biological replicates); asterisks indicate significant differences between transgenic lines and WT (Dunnett’s one-way test; *α* = 0.05), *P*-values indicate the significance of genotype in ANOVA.


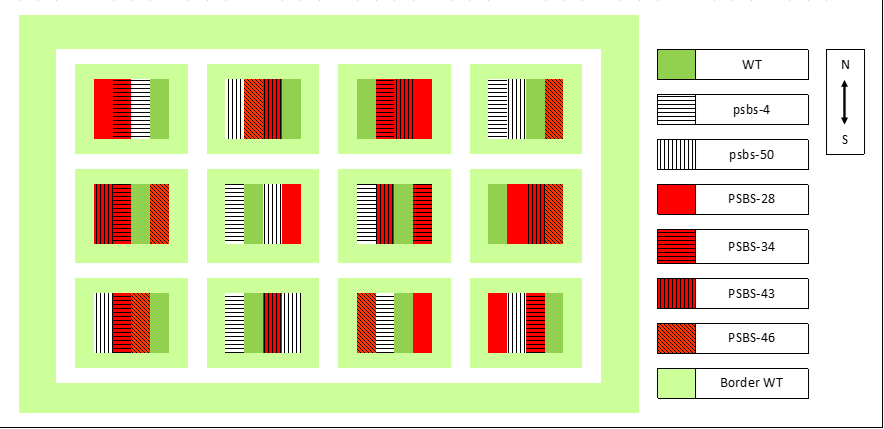


**Supplementary Fig. 4 Schematic representation of field experimental design.**

The field experiment was set up according to a randomized incomplete balanced block design. Each PsbS overexpression and silencing line was randomly assigned one of four possible row positions in six out of 12 blocks, whereas tobacco wild-type (WT) was present in all blocks. Red colored rectangles depict genotypes with increased PsbS expression (PSBS-28, -34, -43, -46); white rectangles represent genotypes with decreased PsbS expression (psbs-4 and -50), and green rectangles and border represent WT.


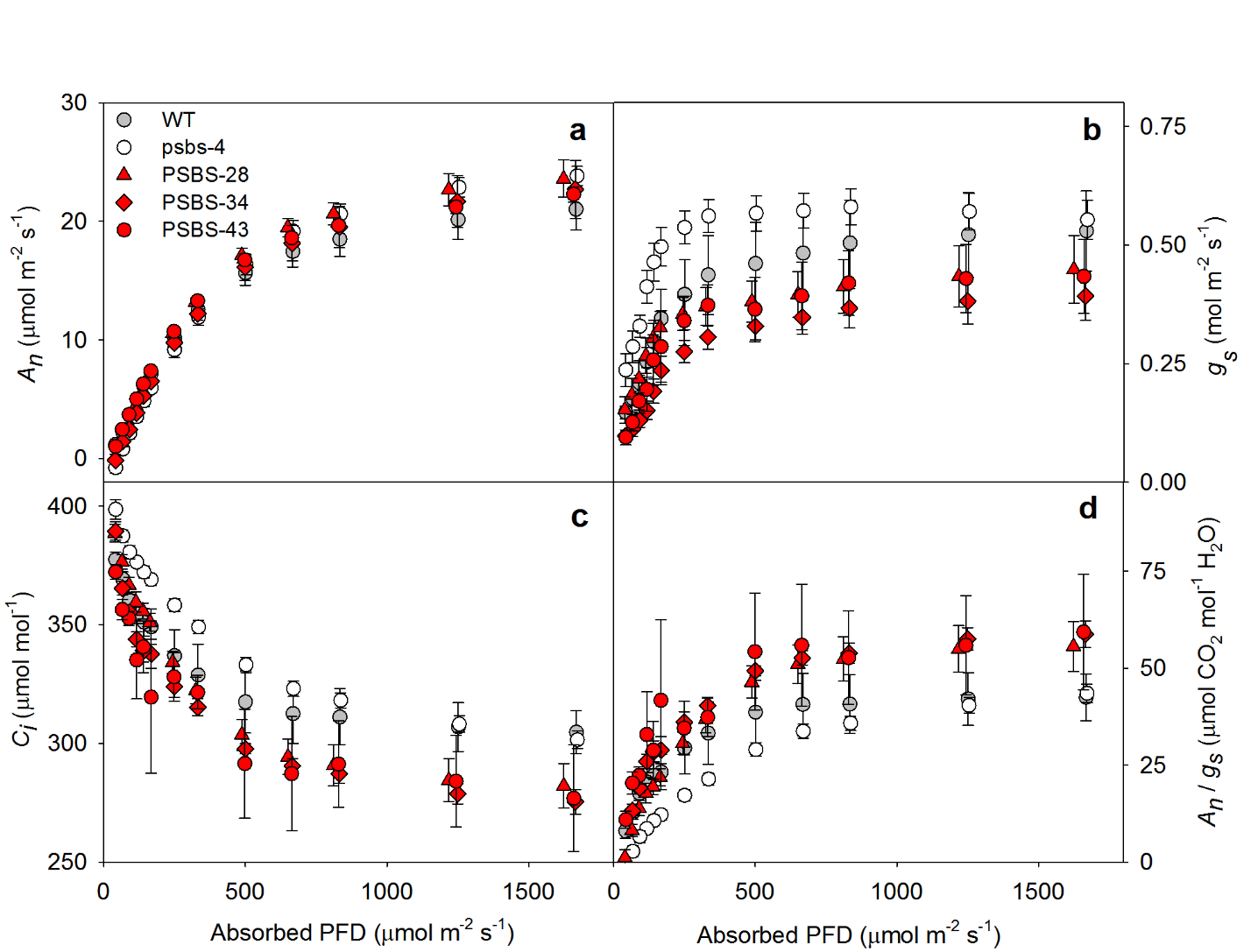


**Supplementary Fig. 5 Response of gas exchange parameters to absorbed light intensity in field-grown *N. tabacum* plants of with modified PsbS levels.**

**(a)** Net CO_2_ fixation rate (*A_n_*), **(b)** Stomatal conductance (*g_s_*), **(c)** Intercellular CO_2_ concentration (*C_i_*), and **(d)** Intrinsic water-use efficiency (*A_n_* / *g_s_*) as a function of absorbed light intensity (PFD) in field-grown plants of PsbS-silencing lines (psbs-4) and PsbS-overexpressing lines (PSBS-28, -34, and -43), and wild-type (WT) *N. tabacum*. Error bars indicate s.e.m. (*n* = 4 biological replicates). Data corresponds to genotype effects shown in Fig. 3a-b of the main manuscript.


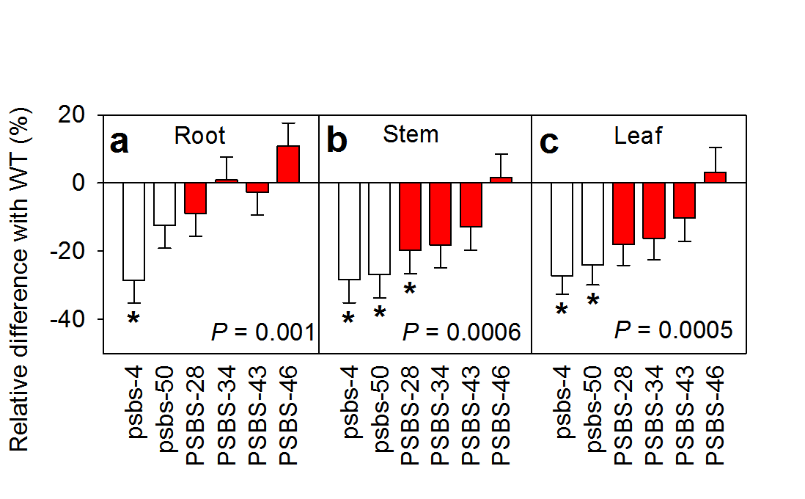


**Supplementary Fig. 6 Leaf, stem and root dry-weight at the end of the field experiment.**

**(a)** Root, **(b)** Stem, and **(c)** Leaf dry-weight at final harvest. Error bars indicate s.e.m., *n* = 6 blocks for transgenic and *n* = 12 blocks for WT), and asterisks indicate significant differences between transgenic lines and WT (Dunnett’s two-way test; *α* = 0.05), *P-*values indicate significance of line effect in ANOVA.


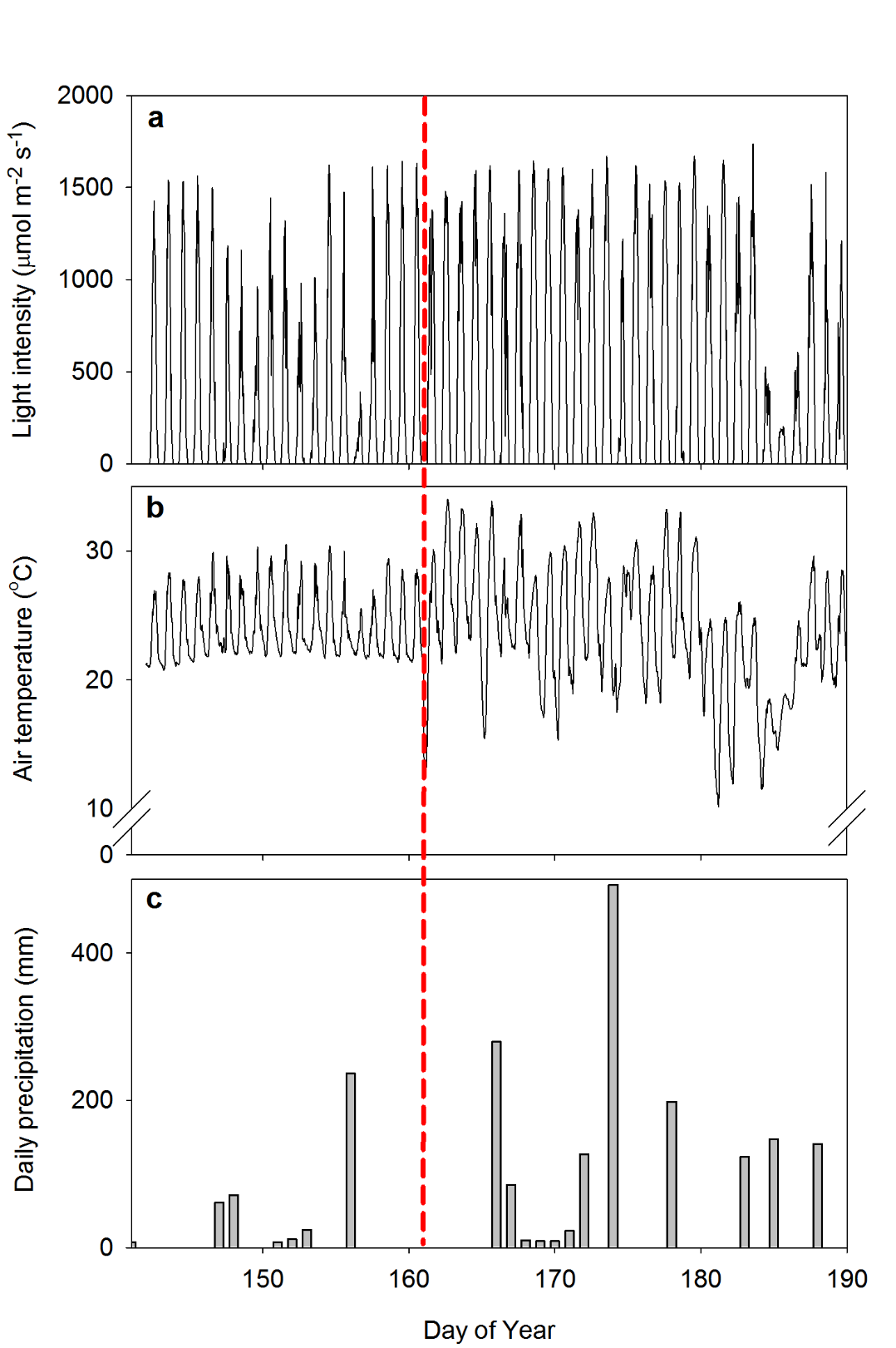


**Supplementary Fig. 7 Weather conditions during propagation and field experiment.**

**(a)** Light intensity from May 21 until July 7, 2016. **(b)** Air temperature from May 21 until July 7, 2016. (**c)** Precipitation from May 21 until July 7, 2016. Data were collated into 30 min averages (**a-b**) or daily totals (**c**). Dashed red line indicates date of field transplant (June 9, 2016). Light intensity and temperature data before transplant were obtained from greenhouse sensors.
